# Non-adjuvanted interferon-armed RBD protein nasal drops protect airway infection from SARS-CoV-2

**DOI:** 10.1038/s41421-022-00411-4

**Published:** 2022-05-10

**Authors:** Yifan Lin, Jing Sun, Xuezhi Cao, Xiuye Wang, Xi Chen, Hairong Xu, Jincun Zhao, Yang-Xin Fu, Hua Peng

**Affiliations:** 1 Institute of Biophysics, Chinese Academy of Sciences, Beijing, China; 2 State Key Laboratory of Respiratory Disease, Guangzhou Institute of Respiratory Health, the First Affiliated Hospital of Guangzhou Medical University, Guangzhou, Guangdong China; 3 Guangzhou Laboratory and Bioland Laboratory, Guangzhou, Guangdong China; 4 LivzonBio Inc., Zhuhai, Guangdong China; 5 Department of Basic Medical Sciences, School of Medicine, Tsinghua University, Beijing, China

**Keywords:** Innate immunity, Biological techniques

Dear Editor,

The COVID-19 pandemic caused by SARS-CoV-2, highly transmissible from person to person via droplets and small particles inhaled in the upper respiratory tract, leads to drastically increased morbidity and mortality globally^1^. Meanwhile, the most recent variant of concern, Omicron, has a substantial growth advantage over Delta in terms of transmission^2^. All WHO-approved vaccines fail to diminish the Omicron variant’s transmission^3,4^. Those vaccines are all administered via intramuscular (i.m.) injection, which induces a high level of serum IgG, but no detectable mucosal IgA in serum and airway^5^. Studies based on monoclonal antibodies suggest that the antibody response produced by i.m. vaccination is insufficient to protect the nasal mucosa^6^, because it is the secreted IgA in the upper respiratory tract, not serum IgG, that dominates the first line of defense to SARS-CoV-2 infection^7^.

Intranasal (i.n.) vaccines can induce airway IgA and reduce viral load at the very early stages of viral infections^8^. Therefore, nasal delivery of safe and potent vaccines should be considered for generating airway mucosal immunity, such as IgA, to reduce respiratory transmission of SARS-CoV-2 rapidly. Effective mucosal delivery of mRNA vaccines has not yet been developed. Most of the current i.n. vaccines, such as adenovirus vector vaccines, rely on infection and show effective mucosal protective responses in preclinical experiment^9–11^. However, the pre-existing immune response against adenoviruses prevalent in most populations could interfere with the efficacy of such viral vector vaccines and the subsequent booster immunization^12^. In addition, the side effects of active infection by viral vector vaccines through i.n. delivery should not be overlooked^13^. Hence, it is necessary to develop novel vaccines for nasal administration^14^.

Recombinant protein vaccines are safe, but with too weak immunogenicity to be employed in airway immunization without adjuvants. Our previous study reported on a fusion-protein vaccine containing the receptor-binding domain (RBD) of the SARS-CoV-2 spike protein and a mouse Interferon, IFNα-Pan-RBD-Fc (IPRF). It is also known as V-01 with human IFNα instead of mouse IFNα applied in monkey studies and clinical trials. In preclinical studies, this interferon-armed RBD protein vaccine induces robust immune responses via i.m. injection even without adjuvant^15^, suggesting that the V-01 vaccine could be a potential effective i.n. vaccine candidate for SARS-CoV-2. To test the immunogenicity of IPRF through nasal drops, we first delivered a low dose (10 μg) of the IPRF or the same amount of RBD protein into the mouse subject’s nasal cavity. Similar to the known protein vaccines, the RBD protein vaccine could hardly induce detectable anti-RBD antibodies in the absence of adjuvants (Fig. 1a). However, strong RBD-specific IgG and impressive high titer of IgA antibodies were induced in the serum of the IPRF-vaccinated group, and these high levels of antibodies persisted for a long period of time. We further measured IgA in the nasal mucosa and confirmed that IPRF nasal drops induced a robust RBD-specific IgA response, but not RBD (Supplementary Fig. S1a). Impressively, the enzyme-linked immunoprotect (ELISpot) assay using the RBD peptide library also demonstrated that nasal IPRF could induce a much stronger RBD-specific T cell response than RBD vaccines (Supplementary Fig. S1b).

**Fig. 1:**
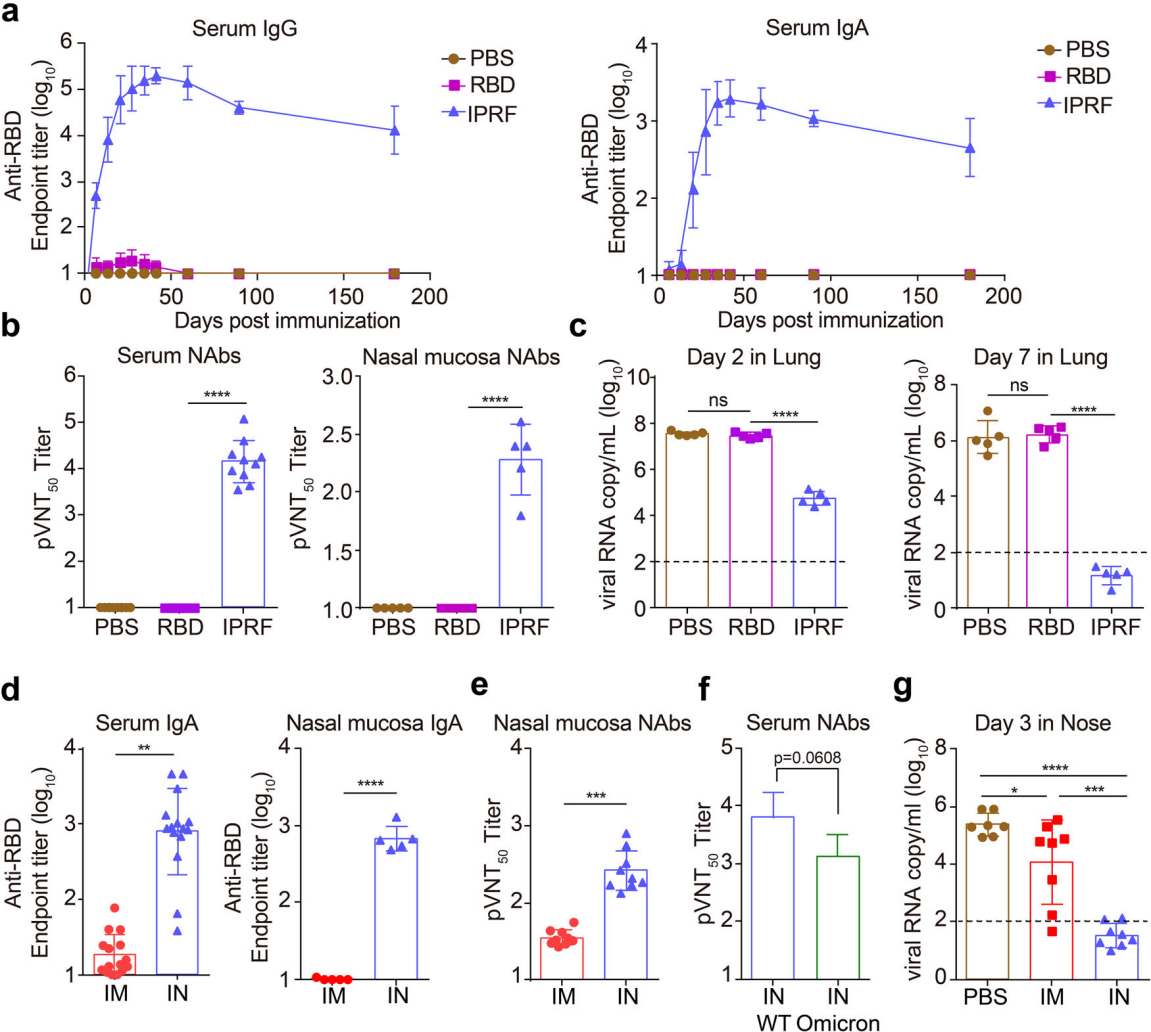
Intranasal administration of IPRF-induced robust IgG and IgA responses against SARS-CoV-2. C57BL/6 mice (*n* = 10) were i.n. immunized with 10 μg IPRF, equal molar of RBD, or PBS. **a** Antibody levels in sera of immunized mice were evaluated weekly after prime and boost vaccination. **b** The viral neutralization antibody titer (NAbs) of vaccinated sera and nasal mucosal collected on day 42 was evaluated using a pseudovirus neutralization assay. **c** The hACE2-transgene mice (*n* = 10) were i.n. immunized with 10 μg IPRF, equal molar of RBD or PBS twice on days 0 and 14. Mice were challenged with authentic SARS-CoV-2 6 weeks post the boost dose. Five mice in each group were euthanized 2 days post challenge. The other five mice in each group were euthanized 1 week post challenge. Viral RNA copies in the lung of each mouse were determined by qRT-PCR and plotted as log10 copies per mL. **d**–**f** C57BL/6 mice (*n* = 10) were i.n. or i.m. immunized with 10 μg IPRF. **d** RBD-specific IgA response in sera and nasal mucosal homogenate supernatant on day 42 was measured by ELISA. **e** Vaccinated mouse nasal mucosal collected on day 42 was evaluated using a WT pseudovirus neutralization assay. **f** The neutralization activity was evaluated using an Omicron pseudovirus neutralization assay. **g** The hACE2-transgene mice (*n* = 8) were i.n. or i.m. immunized with 10 μg IPRF or PBS twice on days 0 and 14. Mice were challenged with authentic SARS-CoV-2 12 weeks post the boost dose. Mice in each group were euthanized 3 days post challenge. Viral RNA copies in the nasal of each mouse were determined by qRT-PCR and plotted as log10 copies per mL. The data shown are presented as mean ± SEM. *P* values were determined by one-way ANOVA with multiple comparison tests. ns, not significant; *P < 0.05, **P < 0.01, ***P < 0.001, ****P < 0.0001.

Robust neutralizing antibodies were also induced by i.n. immunization in both the serum and the supernatant of the nasal mucosa, tested with the original SARS-CoV-2 pseudovirus assay (Fig. 1b). To assess the efficacy of the IPRF i.n. vaccine in preventing airway infection, hACE2-transgenic mice were i.n. immunized and challenged with live SARS-CoV-2 42 days after the initial vaccination. Again, the IPRF i.n. vaccine induced robust anti-RBD IgG and IgA antibody responses, while the RBD vaccine group did not induce a detectable antibody response (Supplementary Fig. S2a, b). Sera from IPRF groups present a significantly higher neutralizing antibody titer against SARS-CoV-2 than the RBD group tested by focus-reduction neutralization test (Supplementary Fig. S2c). We analyzed the viral load and pathological sections by hematoxylin-eosin staining. Compared to the RBD group, IPRF vaccination resulted in a much lower viral load in lung tissue after infection, and no significant inflammatory response was observed in the lung tissue on day 2 after viral challenge in the IPRF group (Fig. 1c and Supplementary Fig. S3). Impressively, the lungs of IPRF-vaccinated mice were cleared of the virus on day 7 post infection (Fig. 1c) and presented no pathology (Supplementary Fig. S3). In contrast, the RBD vaccine failed to reduce viral load and exhibited severe lung pathology. It suggests that conventional protein vaccines without adjuvant might not be able to generate protective immunity.

We next characterized the immune response induced by nasal drops of non-adjuvanted IPRF compared to i.m. injection and found that both administrations induced robust RBD-specific IgG response (Supplementary Fig. S4a). However, only i.n. administration induced a robust RBD-specific IgA antibody response, but not in the i.m. injection group (Fig. 1d). Meanwhile, pseudoviruses were used to evaluate the neutralization titer. There was no significant difference between the viral neutralization titers of sera from mice vaccinated via either IPRF i.m. or i.n. administration (Supplementary Fig. S4b). It is crucial that i.n. drops can induce neutralizing antibodies in the nasal mucosa, while i.m. administration cannot (Fig. 1e). Most impressively, sera from i.n. non-adjuvanted two-dose V-01 vaccinations maintained as strong viral neutralization titers as i.m vaccine against the Omicron pseudovirus (Fig. 1f and Supplementary Fig. S5a). To further compare the anti-viral immunity in the upper respiratory tract induced by i.m. vs i.n. administration, three treatment groups of ACE2 mice (i.m., i.n. vaccination, or PBS) were challenged with authentic SARS-CoV-2 on day 84 after the initial immunization (Supplementary Fig. S6a). We found that on the third day after the viral challenge, the viral load in the lungs decreased significantly in all groups (Supplementary Fig. S6b). Intriguingly, the viral load was not detectable in the nasal mucosa of i.n. vaccinated mice but remained at high levels in the control and i.m. injection groups (Fig. 1g). This result further supports our hypothesis that the nasal route can generate better protection. Indeed, nasal drop vaccination achieves adequate protection, eliminating the virus in the upper respiratory tract and preventing viral spreading in the early stage of infection. Furthermore, an i.m. vaccine may confer relatively weak protection against viral infection in the upper respiratory tract. In the clinic, nasal drops may be a convenient and efficient route of mucosa vaccination, and nasal spray or atomization inhalation should be evaluated for immunization efficiency in future preclinical and clinical trials.

While protein antigens, such as RBD, fail to induce IgA through i.n. vaccination, our study clearly demonstrates that our RBD fusion-protein (IPRF) nasal drops could induce high titers of RBD-specific IgA in the upper respiratory tract without additional adjuvant. Most importantly, this newly designed nasal drop vaccine can stimulate the production of potent RBD-specific neutralizing antibodies in systemic circulation and in the upper respiratory tract to protect mice from SARS-CoV-2 infection.

## Supplementary information

Supplementary Information
