# Supplementary material for: Non-adjuvanted interferon-armed RBD protein nasal drops protect airway infection from SARS-CoV-2
Source: Cell Discov. 2022 May 10;8:43. doi: 10.1038/s41421-022-00411-4 (PMC9089296; doi:10.1038/s41421-022-00411-4)
Supplement: Supplementary file 1 — Supplementary Information [file 41421_2022_411_MOESM1_ESM.pdf]

**Supplementary information**

# A non-adjuvanted interferon-armed RBD protein nasal drops protect airway infection from SARS-CoV-2

Yifan Lin[1,6], Jing Sun[2,6], Xuezhi Cao[3,6], Xiuye Wang[3], Xi Chen[4], Hairong Xu[1], Jincun Zhao[2,3*], Yang-Xin Fu[5,7*] and Hua Peng[1,3*]

**Contents**

**Materials and methods**

**Supplementary figures and legends**

## Methods and Materials

### Animal immunization and sample collection

The recombinant protein antigens were diluted with PBS for mouse immunization. C57BL/6 mice obtained from Vital River (Beijing). For intramuscular administration, mice were immunized intramuscularly with antigen in 100μL using insulin syringes. For intranasal administration, mice were fully anesthetized by i.p. injection of a mixture of 300μL 1.25% tribromoethanol and 2.5% 2-butanol, then use a pipette to drop 5μL antigen from each nostril. Serum samples were collected at the indicated time points and used to perform the antibody assays, and nasal mucosa samples were collected after heart perfusion.

### Heart perfusion

Mice were fully anesthetized by i.p. injection of a mixture of 300μL 1.25% tribromoethanol and 2.5% 2-butanol; then, thoracotomy was performed. The right atrium was cut, then the left ventricle was injected with 100ml PBS by the blunt perfusion needle till all the major organs were white.

### Nasal mucosa tissue collection

After heart perfusion, the intact nasal mucosa and part of the skeleton surrounding it were collected. The supernatant was collected after digestion with collagenase and DnaseI.

### Authentic SARS-CoV-2 infection

The hACE2 mice were purchased from Gem Pharmatech Co, Ltd. and vaccinated as C57BL/6 mice. Mouse sera were collected weekly post the initial vaccination. Mice were challenged with authentic SARS-CoV-2 four weeks post the boost dose. Mice were lightly anesthetized with isoflurane and inoculated intranasally with $4x10^4$ FFU of SARS-CoV-2 viruses. Five mice in each group were euthanized two days post challenge, and the other five in each group were euthanized one week after the challenge. The lungs were collected for RT-PCR and histopathology analysis. Nasal mucosa and bone surrounding it was collected for RT-PCR.

**ELISA**

The 96-well ELISA plates (Corning, USA) were coated with 100 μL of wild-type RBD or variant RBD (1.5μg/mL) overnight at 4°C. Plates were washed with PBS and blocked with blocking buffer (PBS containing 5% fetal bovine serum, FBS). Serum samples were serially diluted and added to the blocked plates, followed by incubation at 37°C for 1 hour. Plates were then washed with PBST (PBS containing 0.05% Tween 20) and incubated with goat anti-mouse IgG-HRP (1:5000, Cwbiotech) or goat anti-mouse IgA (1:2000, Abcam) at 37°C for 30 minutes. Plates were washed with PBST, and HRP substrate TMB was added. The reactions were stopped by 2M sulfuric acid. The absorbance at 450-630 was read using a microplate reader (Molecular Devices). The endpoint titers were defined as the reciprocal of maximum serum dilution at which the absorbance was higher than 2.5-fold of the background.

**Enzyme-linked immunospot (ELISpot) assay**

Murine IFN-γ ELISpot assays were carried out according to the manufacturer's protocols for mouse IFN-γ ELISpot kit (BD Bioscience). Immunized mice splenocytes were seeded in the plates with a density of $2 \times 10^5$ cells per well and incubated with the peptide pool of 15-mer peptides with 11 overlapping amino acids for SARS-CoV-2 RBD protein (5μg/mL) in pre-coated 96-well ELISpot plates with Concanavalin A (Con A, Sigma) as a positive control or medium as a negative control for 48 hours at 37°C. Then, the cells were removed, and biotinylated IFN-γ (BD Bioscience) was added to the plates, followed by incubation for 2 hours at room temperature. The plates were washed three times with PBST before adding Streptavidin-HRP (BD Bioscience). The BD ELISPOT AEC substrate (BD Bioscience) was used to develop the spots. Spots were counted and analyzed using an automated ELOSpot reader (Cellular Technology).

**Pseudovirus neutralization assay**

The pseudovirus neutralization was carried out as described previously. In brief, the pseudovirus was produced by co-transfection of the plasmid expressing firefly luciferase (pNL43R-E-luciferase) and pcDNA3.1 expressing the SARS-CoV-2 spike protein and Omicron variant spike protein into 293T cells. After 48 hours, the viral supernatant was collected, and viral titers were determined by luciferase activity in relative light units. To evaluate the neutralization of vaccinated mice serum, 293-hACE2 cells were seeded into 96-well plates ($2x10^4$ per well), and 3-fold serially

diluted heat-inactivated serum samples were incubated with 100 $TCID_{50}$ of pseudovirus for 1 hour at 37°C. Medium mixed with pseudovirus was given as control. The mixture was transferred to the 96-well plates, and the plates were incubated for another 24 hours. According to the manufacturer's instruction, the luciferase substrate was added, and luciferase activity was determined by the Bright-Lite™ Luciferase Assay System (Vazyme). The 50% neutralization titer ($pVNT_{50}$) was defined as the reciprocal of serum dilution at which the relative light units (RUL) were reduced by 50% compared to the virus control wells.

**Quantitative reverse transcription-polymerase chain reaction (qRT-PCR)**

Viral RNA in lung tissues and nasal mucosa was determined by quantitative reverse transcription PCR (qRT-PCR). In brief, lung tissues were homogenized, and RNA was extracted with a RNeasy Mini kit (QIAGEN). The viral RNA copies were determined using THUNDERBIRDTM probe one-step qRT-PCR kit (TOYOBO) with the following primers and probes: forward primer 5'-GGGGAACTTCTCCTGCTA GAAT-3', reverse primer 5'-CAGACATTTTGCTCTCAAGCTG- 3', and probe FAM-TTGCTGCTGCTTGACAGATT-TAMRA-3'. SARS-CoV-2 RNA reference standard (National Institute of metrology, China) was serially diluted and performed to generate the standard curve.

**Focus Reduction Neutralizing Test (FRNT)**

Vero-E6 cells were seeded. into 96-well plates with a density of $2x10^4$ per well. Sera from immunized animals were serially diluted and mixed with 75 μL of authentic SARS-CoV-2 ($8\times10^3$ focus-forming units (FFU)/mL)). The mixture was incubated for 1 hour at 37°C and then transferred to the 96-well plates seeded with Vero E6 cells. Plates were incubated for 1 hour at 37°C. The inoculums were removed, and the plates were overlaid with medium (100μl DMEM containing 1.6% carboxymethylcellulose, CMC). The plates were incubated for another 24 hours at 37°C. The supernatant was removed, and cells were fixed with 4% Paraformaldehyde for 30 minutes. Cells were subsequently permeabilized with PBS containing 0.2% Triton X-100. After PBS washing three times, the cells were incubated with cross-reactive rabbit anti-SARS-CoV-2 nucleocapsid IgG (Sino Biological) for 1 hour at 37°C. After incubated with the primary antibody, plates were washed three times with PBST before adding the second antibody, HRP-conjugated goat anti-rabbit IgG (Jackson ImmunoResearch). Cells were

further incubated for 1 hour at 37°C. After washing, the KPL TrueBlue peroxidase substrates (Seracare Life Science) were added to the plates. The supernatant was removed, and the plates were washed three times with deionized water five minutes later. The numbers of SARS-CoV-2 foci were read using an ELISpot reader (Cellular Technology). The $FRNT_{50}$ was defined as the sera dilution at which neutralization antibodies inhibited 50% of the viral infection.

**Histopathology**

SARS-CoV-2-challenged hACE2 mice were euthanized in the BSL-3 facility. Mouse lungs were collected and fixed in 4% paraformaldehyde buffer for 48 hours, followed by embedding with paraffin. The sections (3-4 mm) were stained with hematoxylin and eosin (H&E). Images were captured with Pannoramic MIDI.

**Statistical analyses**

All statistical analyses were performed using Graphpad Prism 8.0. Data are shown as the geometric mean with 95% confidence intervals. Statistical analyses were compared with an unpaired or paired student's two-tailed t-test for comparison between two groups. One-way ANOVA with Turkey's multiple comparison test was used for comparisons among multiple groups. P values of $< 0.05$ were considered significant. $p<0.05$ (*), $p<0.01$ (**), $p<0.001$ (***) and $p<0.0001$ (****). ns, no significance.

**Supplementary figures**

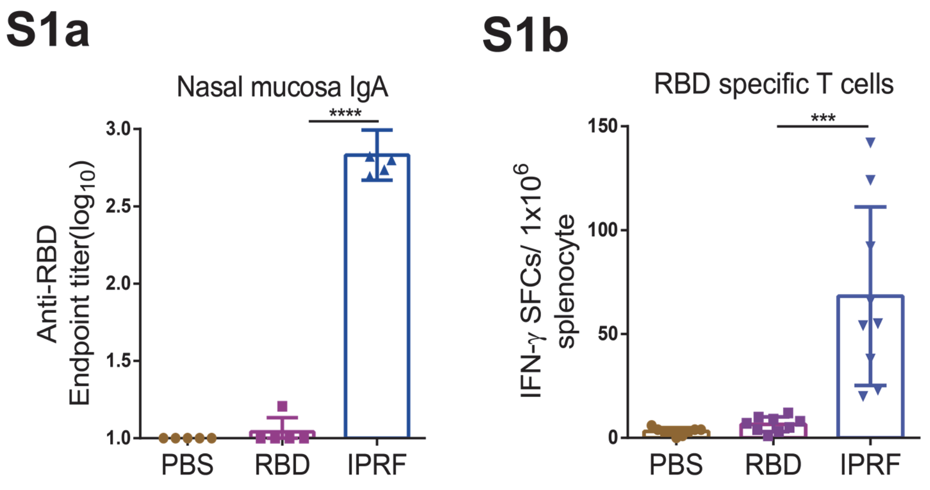


**Figure S1. Nasal mucosal RBD-specific IgA titer and the RBD-specific T cell response induced by IPRF intranasal vaccination.**

(**a**) C57BL/6 mice were i.n. vaccinated with 10 μg I-P-R-F, equimolar RBD, or PBS with a prime-boost vaccination regimen in a 14-day interval. SARS-CoV-2 RBD-specific IgA response in nasal mucosal homogenate supernatant on day 42 post the initial vaccine was analyzed by ELISA. (**b**) Mice were sacrificed, and splenocytes were collected 28 days post the first vaccination. ELISpot assay was performed for IFN-γ secretion from mouse splenocytes stimulated with an RBD peptide pool. Splenocytes were incubated with an RBD peptide pool. The data shown are presented as mean ± SEM. P-values were determined by one-way ANOVA with multiple comparison tests. ns (not significant), *P<0.05, **P<0.01, ***P<0.001, ****P<0.0001.

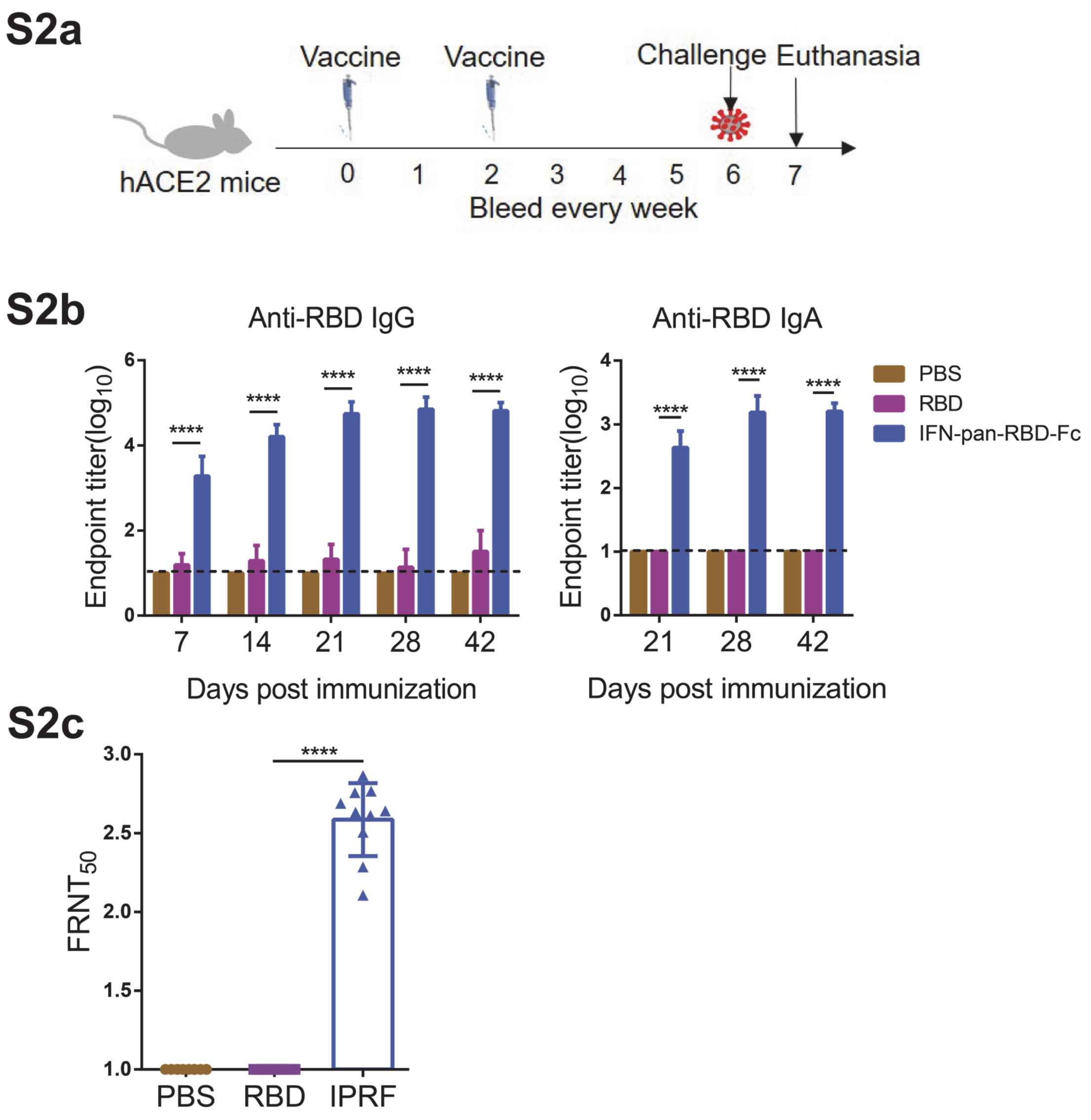


**Figure S2. Live SARS-CoV-2 neutralizing antibody titer in sera from IPRF i.n. vaccinated hACE2 mice.**

(**a**) Schematics of vaccination and viral challenge. The hACE2 transgene mice (n=10) were i.n. immunized with 10μg IPRF, equal molar of RBD or PBS with a prime-boost vaccination regimen in a 14-day interval, and sera were collected weekly. Mice were challenged with authentic SARS-CoV-2 four weeks after the initial boost. Five mice in each group were euthanized two days post the viral challenge. The other five mice in each group were euthanized one-week after the challenge. (**b**) Antibody response in sera of immunized mice was evaluated weekly. (**c**) Serum collected from each mouse was 10-fold serially diluted and incubated with 500 FFU of authentic SARS-CoV-2, then incubated with Vero $10xE^{6}$ cells. The FRNTspots of each well were counted. $FRNT_{50}$ of NAbs of each vaccine group was determined by FRNT and represented as $IC_{50}$, the reciprocal of half-maximal neutralizing dilution. The data shown are presented as mean ± SEM. P-values were determined by one-way ANOVA with multiple comparison tests. ns (not significant), *P<0.05, **P<0.01, ***P<0.001, ****P<0.0001.

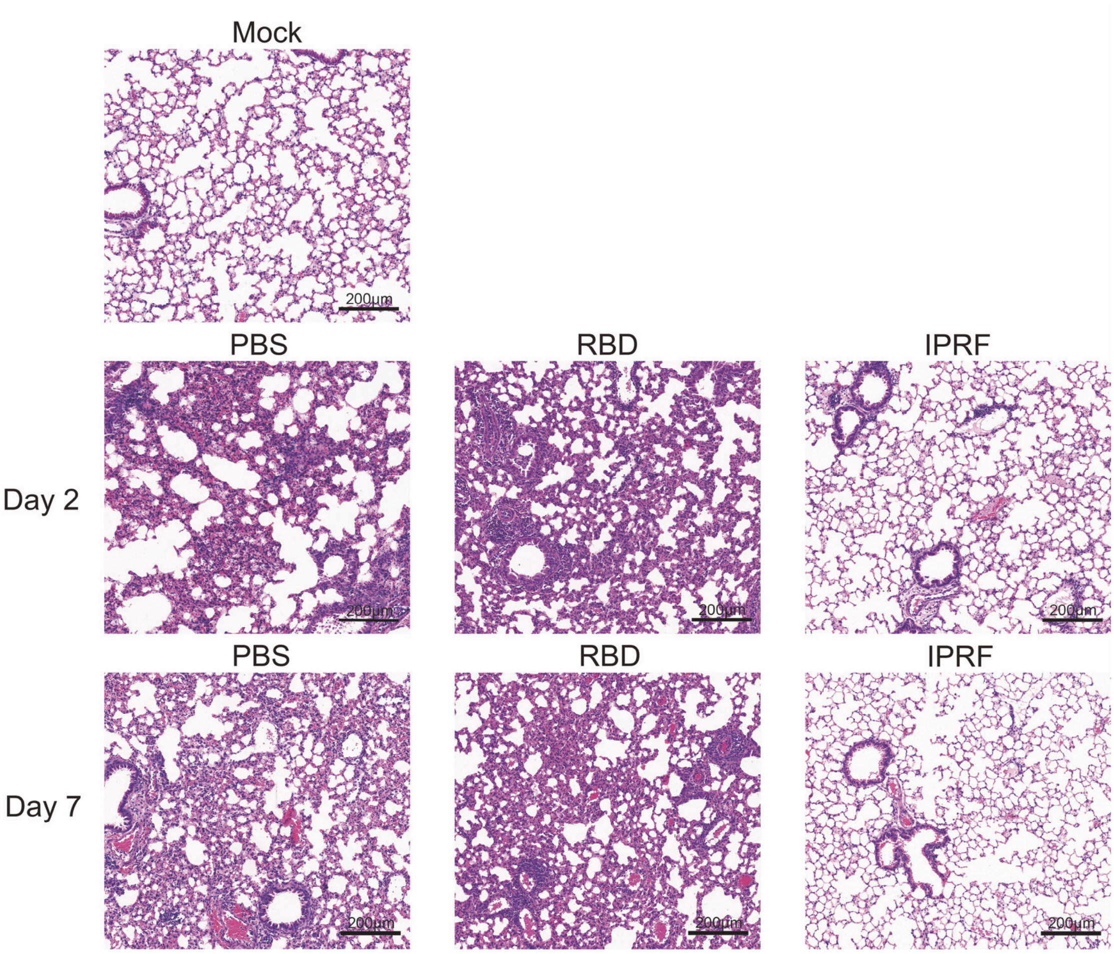


**Figure S3. Inflammation in lung tissues of SARS-CoV-2 infected or uninfected mice**

The hACE2 mice were infected by SARS-CoV-2. The lung tissue sections were collected two days after the SARS-CoV-2 challenge, and the HE staining slides were evaluated for inflammation levels after virus infection.

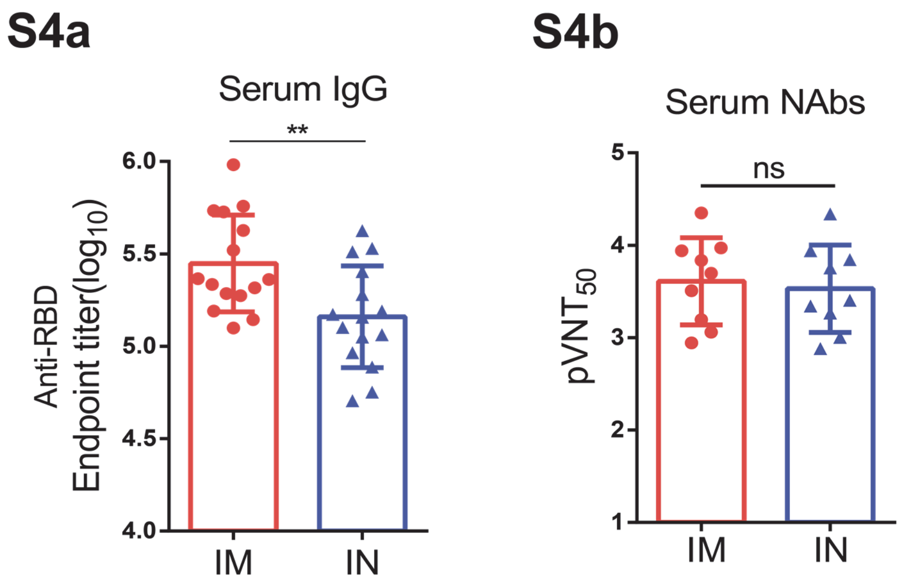


**Figure S4 The RBD-specific IgG and the serum WT SARS-CoV-2 pseudovirus neutralization titer induced by i.m. or i.n. vaccination**

C57BL/6 mice (n=10) were i.n. or i.m. immunized with 10μg IPRF. (**a**) Antibody response in sera of immunized mice was evaluated on day 42 after prime vaccination. Data shown are presented as mean ± SEM. (**b**) NAbs of vaccinated sera collected on day 42 post the initial immunization was evaluated using a pseudovirus neutralization assay. P-values were determined by one-way ANOVA with multiple comparison tests. ns (not significant), *P<0.05, **P<0.01, ***P<0.001, ****P<0.0001.

**S5a**

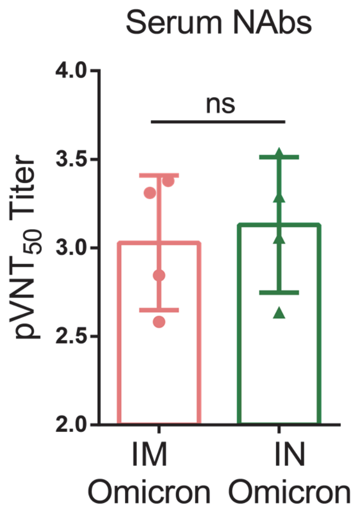


**Figure S5. The serum Omicron SARS-CoV-2 pseudovirus neutralization titer of mice receiving i.m. or i.n. IPRF vaccination**

NAbs of vaccinated sera collected on day 42 post the initial immunization was evaluated using a pseudovirus neutralization assay. The data shown are presented as mean ± SEM. P-values were determined by one-way ANOVA with multiple comparison tests. ns (not significant), *P<0.05, **P<0.01, ***P<0.001, ****P<0.0001.

S6a

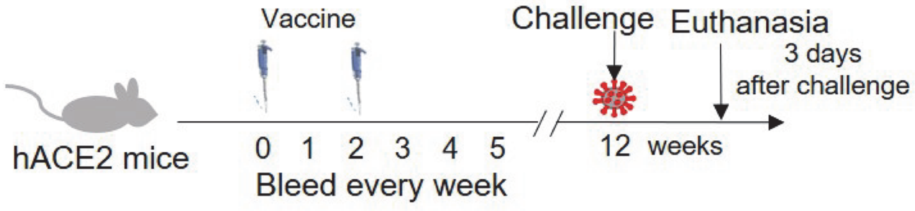


S6b

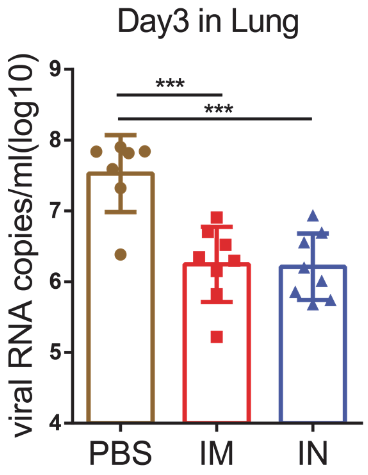


**Figure S6. The WT live SARS-CoV2 neutralization titer of mice receiving i.m. or i.n. IPRF vaccination**

Human-ACE2 transgene mice (n=8) were i.n. or i.m. immunized with 10μg IPRF or PBS following a prime/boost schedule in weeks 0 and 2. Mouse serum was collected weekly. Mice were challenged with authentic SARS-CoV-2 ten weeks post the second immunization and euthanized three days after the viral challenge. (a) Schematics for vaccination and viral challenge. (b) Viral RNA copies in the lungs of each mouse were determined by qRT-PCR and plotted as log10 copies per ml. The data shown are presented as mean ± SEM. P-values were determined by one-way ANOVA with multiple comparison tests. ns (not significant), *P<0.05, **P<0.01, ***P<0.001, ****P<0.0001.
